# Evolution and Key Drivers of Typical Air Pollutants in Binzhou, China: A Case Study of the Yellow River Delta’s Central City (2019–2024)

**DOI:** 10.3390/toxics13121007

**Published:** 2025-11-21

**Authors:** Yan Xu, Jingyu Wen, Mingwei Zhang, Yapeng Li, Yinxiao Zhang, Yueyuan Niu, Xiaotong Jiang

**Affiliations:** 1 Shandong Key Laboratory of Eco-Environmental Science for the Yellow River Delta, Shandong University of Aeronautics, Binzhou 256603, China; 2 Flight College, Shandong University of Aeronautics, Binzhou 256600, China

**Keywords:** PM_2.5_, O_3_, air pollution, temporal variation, source apportionment

## Abstract

In recent years, combined pollution of PM_2.5_ and O_3_ has emerged as a major constraint on improvement of air quality in urban China. This study investigates Binzhou, an industrial–agricultural city within the Beijing–Tianjin–Hebei air pollution transport corridor. Based on air quality monitoring and socioeconomic data from 2019 to 2024, we analyze the temporal variations, driving mechanisms, and economic effects of PM_2.5_-O_3_ compound pollution. Results show that the annual mean PM_2.5_ concentrations decreased initially and then increased, while O_3_ levels exhibited a fluctuating increase. Seasonal patterns were distinct: PM_2.5_ pollution was more severe in autumn and winter, and O_3_ dominated in spring and summer. The number of compound pollution days decreased from 24 in 2019 to 12 in 2024, with a notable concentration in spring (March–May), accounting for 40–54% of the annual total, highlighting this period as critical for coordinated control. Correlation analysis revealed a weak positive association between PM_2.5_ and O_3_ in spring, summer, and autumn (strongest in summer) but a weak negative correlation in winter. Economic development demonstrated a phased decoupling from pollution: Binzhou’s GDP grew by 38.6% cumulatively during the study period, while compound pollution days declined, with significant decoupling in 2020 and 2022. However, pollution rebounded with economic recovery. Key drivers identified include coal combustion and industrial emissions, while industrial restructuring and regional joint prevention policies have contributed to pollution mitigation. This study provides scientific support for formulating differentiated air quality strategies tailored to seasonal and regional characteristics, thereby supporting both clean air and high-quality development.

## 1. Introduction

Under the dual pressures of global climate change and rapid urbanization, synergistic atmospheric pollution, particularly from fine particulate matter (PM_2.5_) and ozone (O_3_), has become as a critical barrier to improving urban environmental quality and public health [1,2]. This challenge is especially acute in heavily polluted regions such as Beijing–Tianjin–Hebei (BTH) and its surroundings, where combined PM_2.5_ and O_3_ pollution poses severe threats to ecological stability and public health [3]. Although the regional collaborative “2 + 26” cities initiative launched in 2017 has significantly reduced PM_2.5_ concentrations through industrial restructuring, emission controls, and joint policies [4,5], substantial spatial and temporal heterogeneity in pollution distribution remains. Certain cities in southern Hebei, Henan, and Shandong provinces—including Binzhou, Dezhou, and Liaocheng—continue to exhibit high PM_2.5_ levels [3]. As a major industrial base in Shandong and a key node in the BTH air pollution transmission corridor, Binzhou significantly influences both local air quality and regional pollutant transport. Therefore, it represents a critical case for investigating composite pollution dynamics.

Existing research shows that the interaction between PM_2.5_ and O_3_ is influenced by seasonal variations, meteorological conditions, and regional transport. For instance, their correlation typically shifts from negative in spring to positive in summer, primarily due to intensified photochemical activity. In winter, this relationship is further influenced by stagnant atmospheric conditions [6]. Regional studies—such as those in the Yangtze River Delta, Fen-Wei Plain, and Jiangxi Province—also reveal significant spatial variations in pollution patterns, governed by meteorological and complex physicochemical processes [7,8,9]. These geographical differences highlight the necessity of region-specific studies. Binzhou exemplifies such regional uniqueness: influenced by heavy industry, geographic location, and agricultural activities, it exhibits a summer pattern dominated by O_3_ (exceedance rate 63.3%) with a distinct inland-increasing spatial gradient, shifting to PM_2.5_-dominated pollution in autumn and winter (exceedance rate ~20%) [10]. This contrasts with other regions, such as the dual spring peaks in Baoding [11] and winter-topography-related amplified PM_2.5_ in the Fen-Wei Plain [12].

However, significant gaps persist in understanding the driving mechanisms and systemic characteristics of PM_2.5_-O_3_ pollution in multi-source cities like Binzhou. Previous studies have predominantly examined megacities and large urban agglomerations. Consequently, the complex interplay of industrial, transportation, agricultural, and meteorological factors in medium-sized urban nodes within air pollution corridors. Quantitative studies in other locales—such as Baoding, where humidity and CO dominate PM_2.5_ and temperature/radiation control O_3_—highlight the region-specificity of key drivers. The role of mobile-source NO_2_ in secondary aerosol formation and ozone competitive chemistry further underscores the need for localized mechanistic analysis [13]. Studies at a smaller urban scale, such as in Yishui County [14], confirm that pollution compositions and drivers can differ markedly from those in larger cities. This evidence reinforces the necessity of tailored studies in underrepresented yet strategically important areas such as Binzhou [15].

Therefore, this study utilizes high-resolution monitoring and socioeconomic data from 2019–2024 to systematically investigate the spatio-temporal evolution, interaction relationships, and multi-dimensional driving mechanisms of PM_2.5_-O_3_ pollution in Binzhou. Using integrated quantitative and qualitative approaches, we evaluate the contributions from local industry, transportation, agricultural emissions, meteorological conditions, and regional transport. A preliminary assessment of environmental and health impacts is also provided. The findings aim to support the design of precise, differentiated control strategies such as optimizing seasonal emission reductions and cross-regional coordination for Binzhou and similar cities. The ultimate goal is to facilitate the synergistic reduction in both pollutants and strengthening regional green development in the Yellow River Basin context.

## 2. Materials and Methods

### 2.1. Data Sources and Processing

The pollutant data used in this study were obtained from the national public environmental data platform (https://www.aqistudy.cn/historydata/monthdata.php (accessed on 10 January 2025)). Daily concentrations of major air pollutants from January 2019 to December 2024 were collected, encompassing a total of 72 observation points (12 months each year). Data quality and measurement procedures comply with the Technical Specification for Ambient Air Quality Index (Trial) (HJ 633-2012) [16]. Seasons were defined according to the Ambient Air Quality Standards (GB 3095-2012) [17]: spring (March–May), summer (June–August), autumn (September–November), and winter (December–February). Socio-economic and emission-related data, including economic indicators and pollutant discharge statistics, were sourced from the Shandong Statistical Yearbook and the Binzhou Statistical Yearbook.

### 2.2. Definition of Compound Pollution

Building on existing research and in accordance with both the Ambient Air Quality Standards (GB 3095-2012) and the WHO Global Air Quality Guidelines (2021), this study defines PM_2.5_-O_3_ compound pollution events. Considering the characteristically low O_3_ concentrations during cold seasons, a day is defined as exhibiting PM_2.5_-O_3_ compound pollution if the daily average PM_2.5_ concentration exceeds 75 μg/m^3^ and the maximum daily 8-h average ozone concentration (O_3_-8 h) exceeds 100 μg/m^3^. Regionally, a compound pollution event is declared when three or more cities within a defined seven-city cluster simultaneously meet the compound pollution criteria on the same day.

### 2.3. Gray Relational Analysis

The GRA method was conducted following the foundational principles of gray system theory originally established by Deng (1982) [18], was employed to assess multi-factor relationships within partially uncertain systems-often denoted as “gray systems”. This method evaluates the degree of similarity between a reference sequence (e.g., a target environmental quality indicator) and comparative sequences (e.g., socio-economic or emission factors) based on the geometric proximity of their data curves. Greater similarity indicates a stronger influence of the factor on the target variable.

The key steps of GRA are described in the following sections.

#### 2.3.1. Data Normalization

Let the reference sequence be $X_{0}=(x_{0}(1),x_{0}(2),\ldots ,x_{0}(n))$ and the comparative sequences be $X_{i}=(x_{i}(1),x_{i}(2),\ldots ,x_{i}(n))$, where i=1,2,…,m. To eliminate dimensional differences, each sequence is normalized using the following formula:

$$x_{i}^{\prime }(k)=\frac{x_{i}(k)-minx_{i}}{maxx_{i}-minx_{i}}$$

#### 2.3.2. Calculation of Gray Relational Coefficient

The gray relational coefficient between $X_{0}$ and $X_{i}$ at the k-th point is calculated as:

$$\gamma (x_{0}(k),x_{i}(k))=\frac{\underset{i}{min}\underset{k}{min}|x_{0}^{\prime }(k)-x_{i}^{\prime }(k)|+\rho \cdot \underset{i}{max}\underset{k}{max}|x_{0}^{\prime }(k)-x_{i}^{\prime }(k)|}{\left|x_{0}^{\prime }(k)-x_{i}^{\prime }(k)|+\rho \cdot \underset{i}{max}\underset{k}{max}|x_{0}^{\prime }(k)-x_{i}^{\prime }(k)\right|}$$

where ρ is the discrimination coefficient, set as 0.5 in this study to balance the influence of extreme values.

#### 2.3.3. Calculation of Gray Relational Grade

The overall gray relational grade between $X_{0}$ and $X_{i}$ is obtained by averaging the relational coefficients:

$$\Gamma (X_{0},X_{i})=\frac{1}{n}\sum_{k=1}^{n}\gamma (x_{0}(k),x_{i}(k))$$

In this study, GRA was applied to quantify the correlations between socio-economic and pollutant emission factors and three categories of environmental quality indicators: ambient air quality, surface water quality, and acoustic environment quality. The input variables for GRA included the following: (1) Reference sequences: concentrations of PM_2.5_, O_3_-8 h, NO_2_, and SO_2_. (2) Comparative sequences: regional GDP (Y1), industrial electricity consumption (Y2), population density (Y3), industrial energy consumption (Y4), agricultural output value (Y5), motor vehicle ownership (Y6), annual precipitation (Y7), and construction area of buildings (Y8). The analysis aims to identify key driving factors and their relative impacts, providing a systematic basis for prioritizing policy interventions.

## 3. Results and Discussion

### 3.1. The Overall Trend of Air Quality Changes in Binzhou City over the Past Six Years

#### 3.1.1. Overall Pollution Changes

**Figure 1:**
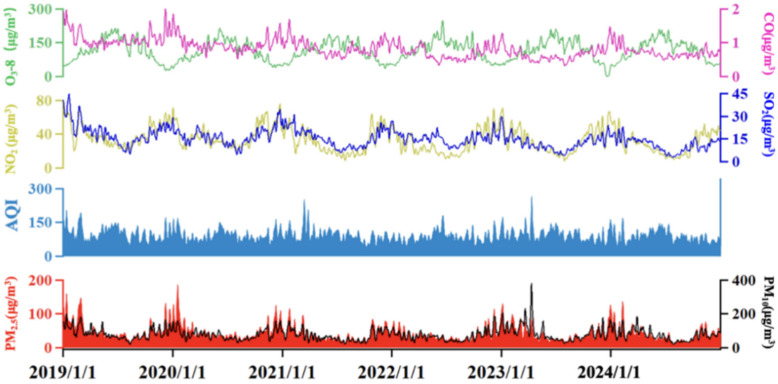
Long-term temporal variations in key air pollutants in Binzhou city from 2019 to 2024.

From 2019 to 2024, air pollution in Binzhou showed a fluctuating trend (Figure 1). A marked decline in NO_2_ concentrations occurred between 2020 and 2021, coinciding with the widespread restrictions on traffic flow and industrial production imposed during the COVID-19 pandemic [19]. These measures directly reduced emissions from key NO_2_ sources like motor vehicles and industrial processes. This finding consistent with studies by Le et al. (2020) [20], who reported similar emission reductions during pandemic lockdowns in other Chinese cities. However, in contrast to the decrease in NO_2_, O_3_ concentrations increased during this period, a phenomenon attributed to enhanced solar radiation and elevated atmospheric oxidizability [21]. This observation supports Kou et al.’s (2023) [21] conclusion that reduced primary pollutant (e.g., NO) can weaken the ozone “titration effect” (NO + O_3_ → NO_2_ + O_2_), thereby promoting O_3_ accumulation even under emission control measures.

During the pandemic, restrictions on industry and transport significantly reduced PM_2.5_ emissions, resulting in lower concentrations. However, after economic activities resumed in late 2022, NO_2_ and O_3_ concentrations rebounded due to increased anthropogenic emissions-particularly of O_3_ precursors (NO_X_ and VOCs). PM_2.5_ concentrations also rose sharply in 2023 but fell in 2024 following targeted pollution control policies such as the “Blue Sky Project.” This trend highlights the effectiveness of policy interventions in mitigating pollution and aligns with Zheng et al. (2018) [22], who highlighted the critical role of clean air actions in preventing post-pandemic pollutant rebound.

To further contextualize the improvements in air quality, Table 1 reveals a gradual increase in the proportion of “Good” air quality days from 2019 to 2022, alongside a decrease in “Unhealthy for Sensitive Groups” “Very Unhealthy” and “Hazardous” days. This trend aligns with Giani et al.’s (2023) [23], who reported short-term air quality benefits from pandemic-related emission reductions. From 2022 to 2024, the proportion of “Good” days fluctuated but stabilized overall. However, “Unhealthy” days temporarily rebounded in 2023, likely because of resumed industrial production. Notably, the lack of a significant deterioration in 2024 highlights the success of Binzhou’s long-term environmental governance measures, including regional joint prevention and control [21].

Table 1 quantifies these trends by detailing air quality grades over the six-year period. The data show that “Moderate” and “Unhealthy for Sensitive Groups” days accounted for 51% and 26% of the total, respectively. Meanwhile, heavily polluted days (Unhealthy and above) from 16% in 2019 to 7% in 2024. The number of “Good” days increased by 59% (from 34 to 54 days), a trend that outpaces the national average for industrial cities in the Beijing–Tianjin–Hebei (BTH) corridor [3]. A notable exception occurred in 2023, when “Good” days decreased to 45 (12%), a decline likely caused by increased road dust from post-pandemic transportation recovery. This finding reveals the vulnerability of air quality to rapid economic recovery and underscores the need for sustained control measures, consistent with Li et al.’s (2021) [24] research on the decoupling of economic growth and pollution.

The monthly Air Quality Index (AQI) variation further reveals seasonal air quality patterns (Figure 1). While the overall trend remained stable, AQI values were consistently lower in summer, benefiting from favorable diffusion conditions and precipitation [25]. Conversely, winter exhibited higher AQI levels due to increased coal combustion for heating and stable atmospheric conditions [26]. This seasonal pattern is consistent with observations in other northern Chinese cities, such as Baoding and Urumqi [12,25], but Binzhou’s unique position in the BTH transmission corridor led to more pronounced AQI spikes during regional pollution episodes (e.g., March 2021, AQI = 141). Targeted control measures-including dust control, industrial emission reductions, and mobile source management-contributed to a significant air quality improvement. For instance, PM_2.5_ concentrations reached 39 μg/m^3^ and the “Excellent” air quality rate attained 53.1% in the first eight months of 2023. By 2024, daily pollutant emissions had decreased by over 10% compared to previous years, with a 30% reduction during autumn and winter-a result that demonstrates the efficacy of Binzhou’s differentiated control strategies.

#### 3.1.2. Characteristics of the Pollution Season

**Figure 2:**
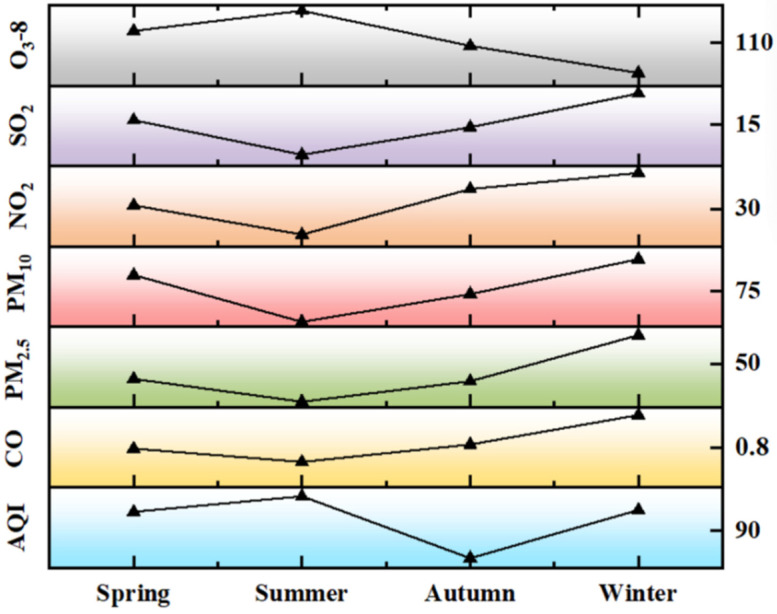
Seasonal variations in different pollutants in Binzhou city from 2019 to 2024.

Compound pollution events in Binzhou showed strong seasonal clustering, with nearly all occurrences concentrated in spring, autumn, and winter, and almost none in summer (Table 2). Spring was the most critical season, accounting for 40–54% of annual compound pollution days-for example, 54% in 2019 and 50% in 2021. This proportion is higher than that observed in other Beijing–Tianjin–Hebei (BTH) corridor cities, such as Baoding, where spring exhibits dual PM_2.5_ and O_3_ peaks [12], or the Fen–Wei Plain, where PM_2.5_ dominates in winter [9]. Figure 2 illustrates these seasonal pollutant variations, and this distinct pattern highlights the need for region-specific springtime control measures in Binzhou.

Autumn compound pollution remained relatively stable (4–7 days/year), while winter days showed a downward trend, stabilizing at 1–4 days after 2020. The primary pollutant also shifted with the seasons: O_3_ dominated in summer (average 159.60 μg/m^3^, far exceeding the national secondary standard of 100 μg/m^3^), while PM_2.5_ was the primary pollutant in autumn and winter. Intense solar radiation and high temperatures in summer promoted photochemical reactions, driving O_3_ formation. This mechanism is consistent with Qin et al.’s (2025), who reported that heatwaves enhance O_3_ formation through temperature-induced VOC emissions [27]. In contrast, PM_2.5_ and PM_10_ concentrations reached their annual minima during summer (27.20 μg/m^3^ and 49.22 μg/m^3^, respectively), due to wet deposition and favorable diffusion [28].

Winter PM_2.5_ concentrations (66.98 μg/m^3^) were 2.46 times higher than in summer, exceeding the national standard by 91%-a result attributed to coal-fired heating and stable atmospheric conditions, mechanism also extensively documented in the severe haze episodes over central and eastern China [29]. Winter also recorded the highest concentrations of SO_2_ (20.37 μg/m^3^) and CO (1.04 mg/m^3^), reinforcing the significant role of fossil fuel combustion. These results are consistent with Wang et al.’s (2014) analysis of winter haze in central and eastern China [29]. However, Binzhou exhibited higher PM_2.5_ levels than those reported in the earlier study, a difference likely attributable to its stronger industrial and agricultural emissions.

Autumn functioned as a transitional period, with PM_2.5_ and NO_2_ concentrations rising and O_3_ declining. The average PM_2.5_ concentration (39.46 μg/m^3^) was slightly lower than in spring. In contrast, NO_2_ levels (38.07 μg/m^3^) were the second highest annually, a pattern likely resulting from increased industrial and transportation emissions coupled with poorer atmospheric dispersion. Meanwhile, O_3_ concentrations (104.60 μg/m^3^) remained above the standard, indicating ongoing photochemical activity despite cooler temperatures.

Spring in Binzhou exhibited a “double high” pattern of PM_10_ and O_3_ pollution. PM_10_ concentrations averaged 88.51 μg/m^3^, a level surpassed only by winter levels, which reflects the influence of frequent dust events. Concurrently, O_3_ concentrations rose to 128.07 μg/m^3^, driven by intensified solar radiation [30]. PM_2.5_ levels (40.84 μg/m^3^) were comparable to autumn, likely due to the secondary pollution of dust and local pollutants [31]. This synergistic PM-O_3_ pollution represents a distinctive feature of Binzhou’s spring, setting it apart from other regions in the BTH corridor.

The seasonal variations in major pollutants followed distinct patterns. PM_2.5_ concentrations were highest in winter (66.98 μg/m^3^), followed by spring (40.84 μg/m^3^) and autumn (39.46 μg/m^3^), and lowest in summer (27.20 μg/m^3^). In contrast, O_3_ showed an opposite trend, peaking in summer (159.60 μg/m^3^) and reaching its lowest level in winter (62.19 μg/m^3^). NO_X_ concentrations were highest in winter (44.69 μg/m^3^) and lowest in summer (19.46 μg/m^3^). Both SO_2_ and CO exhibited pronounced winter peaks (20.37 μg/m^3^ and 1.04 mg/m^3^, respectively), reflecting the substantial impact of coal combustion for heating. PM_10_ levels were highest in winter (101.79 μg/m^3^), with elevated spring concentrations (88.51 μg/m^3^) due to frequent dust events.

The driving mechanisms of O_3_ pollution are strongly influenced by precursor emissions (NO_x_ and VOCs) and meteorological conditions, particularly in spring and summer. Key chemical reactions governing O_3_ formation include the photolysis of NO_2_, which is the primary initiator:

NO_2_ + *hν*(*λ* < 420 nm) → NO + O(^3^P)

O(^3^P) + O_2_→O_3_

NO + O_3_ → NO_2_ + O_2_

However, the resulting O_3_ can be rapidly titrated by NO, forming a null cycle: NO + O_3_ → NO_2_ + O_2_ accumulation occurs only in the presence of volatile organic compounds (VOCs). The hydroxyl radical (OH) initiates VOC oxidation pathways that convert NO to NO_2_ without consuming O_3_:

VOC + OH → RO_2_

RO_2_·+ NO → RO·+ NO_2_

This reaction sequence is critical as it regenerates NO_2_, allowing for further O_3_ production upon its photolysis, thereby leading to net O_3_ accumulation [30]. High temperatures and intense solar radiation in spring and summer act as powerful catalysts for these processes. Elevated temperatures not only accelerate the kinetics of photochemical reactions but also promote the biogenic emissions of highly reactive VOCs, such as isoprene, from vegetation. Furthermore, recent evidence suggests that high temperatures can directly enhance emissions of anthropogenic VOCs, further fueling ozone production, particularly during heatwave events [27] Concurrently, strong solar radiation provides the essential photon flux for NO_2_ photolysis and enhances OH radical concentrations, thereby intensifying the overall oxidative capacity of the atmosphere and facilitating rapid O_3_ production [21].

The sensitivity of O_3_ formation to its precursors exhibits a strong seasonal and regional pattern. During summer, the high baseline levels of NO_x_ in some urban areas often push the photochemical regime from NO_x_-sensitive to VOC-sensitive [32]. In a VOC-sensitive regime, reducing NO_x_ emissions may be ineffective or even lead to a paradoxical increase in O_3_ (a phenomenon known as the “NO_x_ disbenefit effect”), whereas controlling VOC emissions becomes the more effective strategy [33]. This underscores the critical need for targeted VOC control in Binzhou, especially during the high-O_3_ season, to mitigate the escalating ozone pollution.

Overall, most pollutant concentrations peaked in winter and reached their lowest levels in summer. In contrast, ozone (O_3_-8 h) showed an inverse pattern, with its highest concentration in summer, followed by spring, and lower levels in autumn and winter. Consistently, the Air Quality Index (AQI) was highest during winter and lowest in summer. Summer’s intense solar radiation and high temperatures strongly favor ozone formation [27], a process also promoted by the enhanced sunlight characteristic of spring. In autumn and winter, weakened solar radiation weakens, and temperatures suppress photochemical activity, reducing ozone formation [34]. In contrast, SO_2_, NO_2_, PM_10_, PM_2.5_, and CO all peak in winter, followed by autumn, with lower concentrations in spring and summer. This pattern results from increased coal combustion for heating and higher energy demand in winter, coupled with stable atmospheric conditions and frequent temperature inversions that trap pollutants [29]. Autumn exhibits a transitional pattern, with rising emissions from early heating and industrial activity. Conversely, spring and summer benefit from greater precipitation—which removes pollutants through wet deposition—and more favorable conditions for atmospheric dispersion [28]. The Air Quality Index (AQI) mirrors these trends, with the lowest values in summer and the highest in winter. Summer’s generally better air quality stems from lower primary pollutant levels and effective removal by rain and atmospheric mixing. However, high temperatures and strong radiation during this season promote ozone formation via photochemistry [35].

Binzhou’s warm temperate monsoonal climate further modulates these patterns. Spring brings strong, dry winds that aid pollutant dispersion, though increased sunlight also favors ozone production. In autumn and winter, heating and other energy-intensive activities elevate emissions, while cold, dry, and stable atmospheric conditions inhibit dispersion, resulting in higher AQI values.

#### 3.1.3. The Changing Trends of NO_2_ and SO_2_

**Figure 3:**
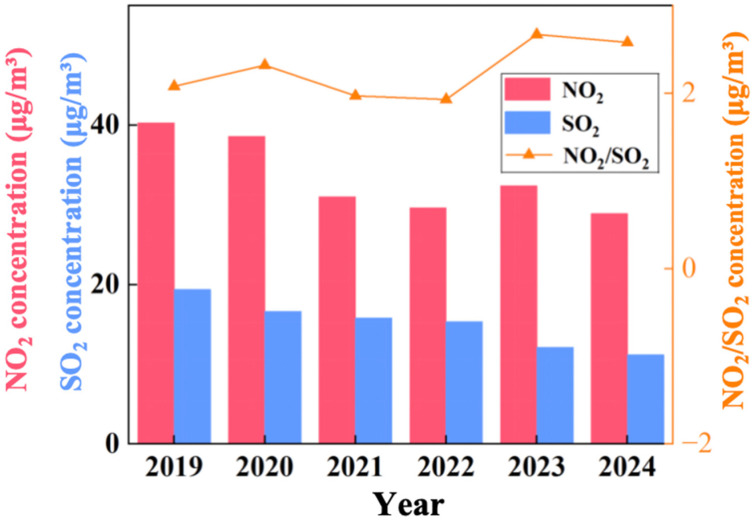
Temporal variations of NO_2_, SO_2_ concentrations and NO_2_/SO_2_ ratio in Binzhou city from 2019 to 2024.

As key indicators of industrial and mobile source emissions, NO_2_ and SO_2_ play critical roles in forming secondary PM_2.5_ and O_3_. Understanding their temporal trends is essential for understanding pollution dynamics. Figure 3 shows a significant decline in SO_2_ concentrations from 19.37 μg/m^3^ (2019) to 11.19 μg/m^3^ (2024), a 42.2% reduction-with the largest drop (21%) occurring in 2023. This decline is attributed to Binzhou’s “coal-to-gas/electricity” projects and the elimination of small coal-fired boilers, which align with national efforts to reduce coal combustion emissions [22]. In contrast, NO_2_ concentrations decreased by 28.3%, from 40.29 μg/m^3^ to 28.89 μg/m^3^, albeit with some fluctuations, and reached a new low in 2024. This decrease is linked to the installation of SCR/SNCR denitrification systems in local industries, a measure also observed in other industrial cities [22].

The NO_2_/SO_2_ ratio increased from 2.08 (2019) to 2.58 (2024), indicating a shift in dominant pollution sources. This trend reflects the growing contribution of mobile sources (which primarily emit NO_2_), relative to industrial coal combustion (a major SO_2_ source). The rising importance of mobile emissions is consistent with the growth in motor vehicle ownership in Binzhou, particularly diesel trucks, as also documented in broader studies of on-road emissions in China [13]. During the pandemic (2020–2021), NO_2_ levels dropped sharply due to traffic restrictions but rebounded after 2022 as economic activity recovered. In contrast, SO_2_ concentrations increased at a slower rate during the recovery phase, reflecting the sustained impact of clean energy policies. This divergence underscores the need for more targeted control measures focused on mobile sources to effectively reduce NO_2_ pollution.

### 3.2. The Current Situation of Combined Pollution of PM_2.5_ and O_3_

#### 3.2.1. PM_2.5_ Concentration Characteristics

**Figure 4:**
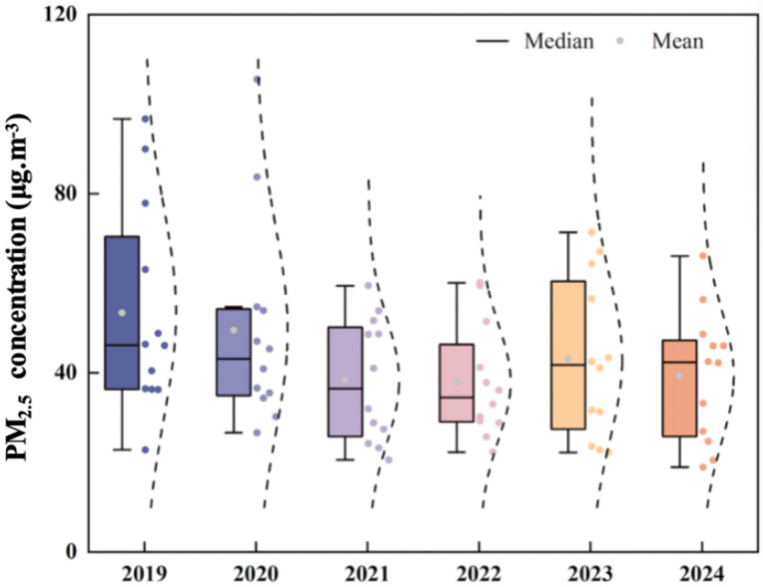
Box plot of PM_2.5_ concentration variation in Binzhou city from 2019 to 2024.

PM_2.5_, a primary pollutant in winter and key component of compound pollution, shows clear temporal variations that are critical for understanding air quality trends in Binzhou. Figure 1 also displays the monthly variation in PM_2.5_ concentrations, with distinct seasonal patterns: higher concentrations in winter (e.g., January 2020, 105.52 μg/m^3^) and lower concentrations in summer. The winter maximum results from coal-fired heating and stable meteorological conditions [29], while the summer minimum is due to wet deposition [28]. Box plot analysis (Figure 4) further indicates a gradual decline in both median and mean PM_2.5_ concentrations from 2019 to 2024. The distribution of data also became more concentrated over this period, reflecting improved stability in air quality. This downward trend aligns with national PM_2.5_ reduction targets [5] but exceeds the average reduction observed in other BTH corridor cities [3], reflecting Binzhou’s effective industrial restructuring and emission controls policies.

PM_2.5_ concentrations in 2021 were lower than in 2019–2020, reflecting the continued influence of pandemic-related emission reductions and the shutdown of highly polluting industries. However, a temporary rebound occurred in 2023, which coincided with increased industrial activity and a 4.2% rise in coal consumption reported in Shandong’s energy statistics. This rebound highlights the persistent challenge of maintaining pollution control during economic recovery-a finding that supports Li et al.’s (2021) [24] research on the decoupling of GDP and pollution.

#### 3.2.2. O_3_ Reacts with Photochemical Reactions

**Figure 5:**
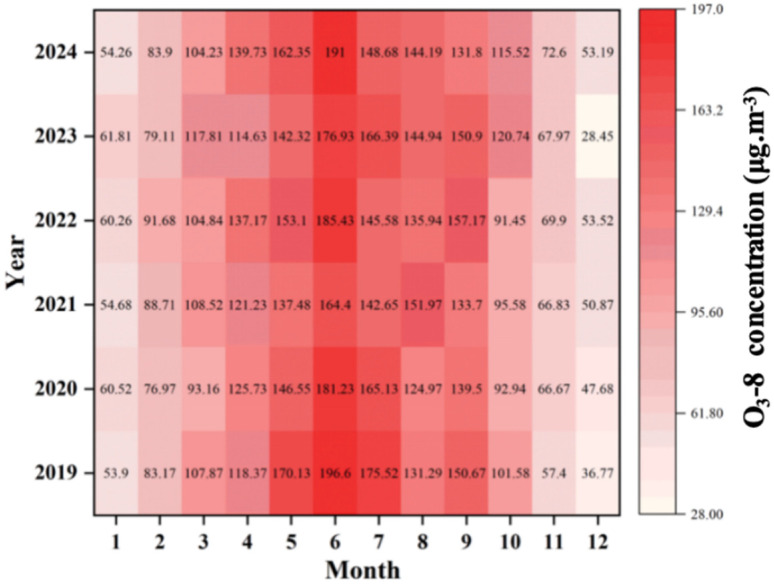
Monthly variation of O_3_ concentration in Binzhou city from 2019 to 2024.

O_3_ serves as the primary pollutant in summer and a major component of spring compound pollution. Its dynamics are strongly influenced by photochemical processes and precursor emissions. Monthly O_3_ concentrations followed a clear pattern (Figure 5), characterized by high pre-pandemic levels, a decline during the pandemic, and a rebound thereafter. Summer peaks-such as 196.6 μg/m^3^ in June 2019 and 185.43 μg/m^3^ in June 2022 were driven by intense photochemical reactions [27]. In contrast, winter minima (e.g., January 2020, 36.77 μg/m^3^) result from weak solar radiation. The pandemic period (2020–2021) saw a temporary O_3_ decline due to reduced precursor emissions, a phenomenon observed across China during the lockdown period [36]. but concentrations rebounded post-2022 with the recovery of industrial and transportation activities. By 2024, O_3_ concentrations remained at a high level, due to abundant precursors (NO_X_ and VOCs) and favorable meteorological conditions-consistent with Qin et al.’s (2025) [27] prediction of increased urban O_3_ in heatwaves.

**Figure 6:**
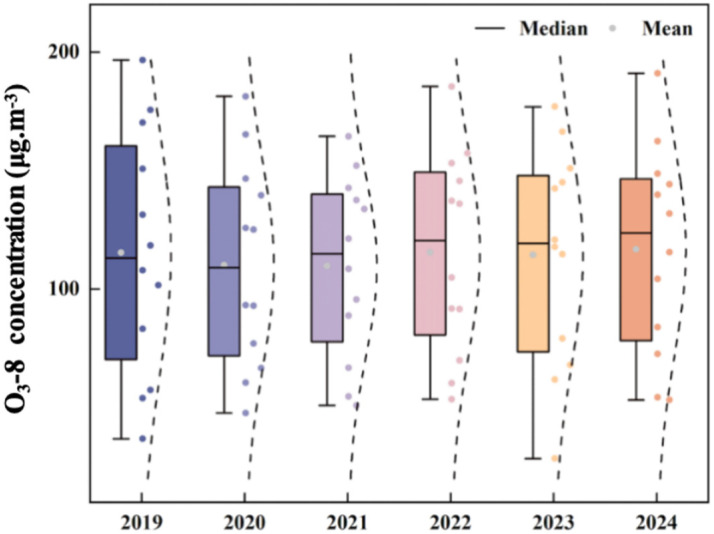
Box plot of O_3_-8 concentration variation in Binzhou city from 2019 to 2024.

The box plot (Figure 6) shows a gradual increase in median and mean O_3_ concentrations from 2019 to 2024. Data dispersion also increased during this period, reflecting greater variability influenced by the interplay of anthropogenic emissions and meteorological conditions. This trend is a cause for concern, as long-term exposure to high O_3_ concentrations increases the risk of respiratory and cardiovascular diseases [37], and the substantial health impacts of persistent air pollution in China are well-recognized [38]. Moreover, Binzhou experiences more severe O_3_ pollution than other BTH corridor cities [3], underscoring the urgency of implementing targeted VOC and NO_X_ control measures.

#### 3.2.3. Combined Pollutions of PM_2.5_ and O_3_

The co-occurrence of PM_2.5_ and O_3_ pollution poses unique challenges for air quality management due to their frequently conflicting control requirements. As shown in Table 3, compound pollution days exhibited a fluctuating but overall decline from 24 days in 2019 to 12 days in 2024, representing a 50% reduction. This decline is attributed to Binzhou’s refined pollution control measures, such as early intervention in heavy pollution events and precise source control [39], consistent with the regional “2+26” cities joint prevention initiative [4]. However, the rebound in 2021 (20 days) and 2023 (14 days) underscores the vulnerability of compound pollution to economic recovery—a finding that complements He et al.’s (2025) [11] observations of PM_2.5_-O_3_ double-high pollution in Baoding during periods of high industrial activity.

**Figure 7:**
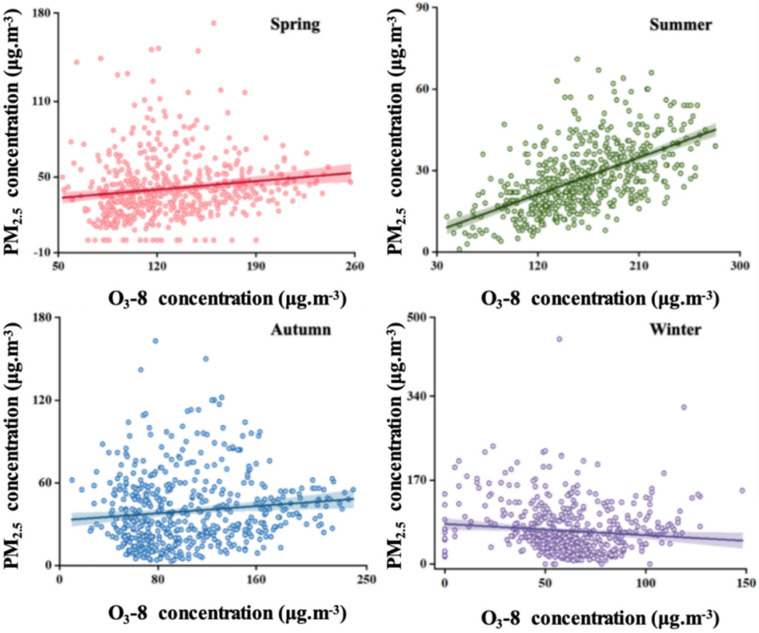
Relationship between PM_2.5_ and O_3_ concentrations in different seasons of Binzhou city from 2019 to 2024.

While conducting the above research, Figure 7 illustrates the seasonal correlation between PM_2.5_ and O_3_: weak positive correlations were observed in spring (r = 0.06), summer (r = 0.15), and autumn (r = 0.09), while a weak negative correlation occurred in winter (r = −0.02). The strongest positive correlation in summer results from vigorous photochemical reactions that generate both O_3_ and secondary PM_2.5_ [27], combined with stable meteorological conditions favoring pollutant accumulation. This finding is consistent with Li et al.’s (2024) [6]. However, the higher summer correlation in Binzhou highlights the additional influence of local industrial emissions.

The weak positive correlation observed in spring arises from competing influences: increased solar radiation promotes O_3_ formation, while dust events introduce exogenous PM_2.5_ [31]. In winter, the negative correlation results from suppressed O_3_ production due to reduced photochemical activity, alongside elevated PM_2.5_ emissions from coal combustion. Although this pattern has been documented in other northern Chinese cities [29], it is less pronounced in Binzhou, likely due to the greater relative contribution of industrial sources to PM_2.5_.

### 3.3. Key Drivers Identified by Gray Relational Analysis

#### 3.3.1. The Characteristics of Compound Pollution Based on the Background of GDP Growth

**Figure 8:**
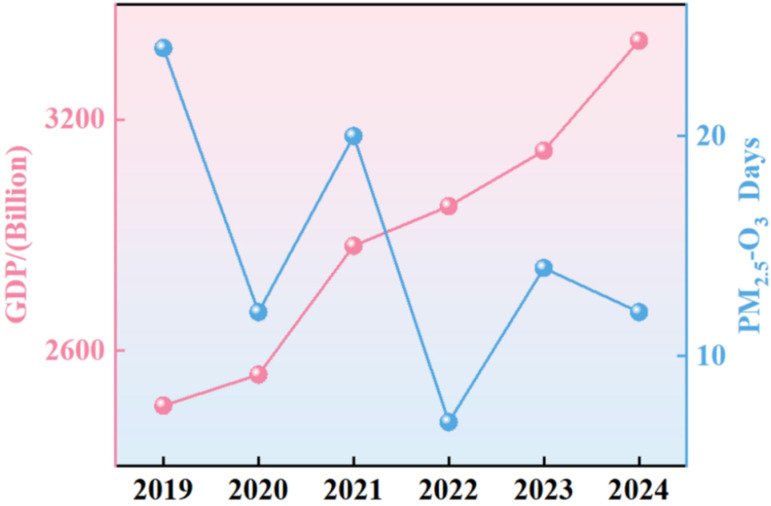
Temporal trends of GDP and PM_2.5_-O_3_ days in Binzhou city from 2019 to 2024.

Understanding the relationship between economic growth and pollution is critical for sustainable development. Figure 8 presents the GDP growth trajectory of Binzhou from 2019 to 2024 alongside the variation in the number of PM_2.5_-O_3_ compound pollution days. The city’s economy grew continuously during this period: GDP increased from 245.7 billion yuan in 2019 to 340.5 billion yuan in 2024, representing cumulative growth of 38.6% and an average annual growth rate of approximately 6.7%. Concurrently, the number of compound pollution days exhibited a fluctuating downward trend, decreasing from 24 days in 2019 to 12 days in 2024-a 50% reduction. This inverse trend suggests a phased “decoupling” between economic growth and air pollution in Binzhou, which aligns with the broader research conclusions of Li et al. (2021) [24] regarding the decoupling relationship between pollutants and GDP in key air pollution control regions of China. It further validates the critical role of targeted environmental policies in facilitating this decoupling process.

Specifically, significant decoupling occurred in 2020 and 2022, with GDP growing by 3.3% and 3.6%, respectively, in these years. In 2022, strict implementation of staggered production measures during autumn and winter effectively counterbalanced the additional pollutant emissions from economic activities. This allowed simultaneous economic growth and substantial pollution reduction, demonstrating how temporary, targeted emission controls can reconcile economic development with environmental protection. These findings provide empirical support for policy-driven decoupling mechanisms [40]. In contrast, weak decoupling characterized in 2021 and 2023. These years saw high economic growth (13.2% in 2021 and 4.8% in 2023) but also notable pollution rebounds, with compound pollution days increasing by 67% and 100%, respectively. The 2023 rebound was partly driven by expanded export manufacturing, which increased energy demand and coal consumption by 4.2% according to Shandong’s energy statistics. The 2021 rebound on the other hand, was primarily attributed to retaliatory industrial production following the relaxation of pandemic restrictions and unfavorable meteorological conditions for pollutant diffusion [20], highlighting the significant economic and environmental pressures posed by the energy-intensive industrial structure [41]. These two distinct decoupling scenarios illustrate the complex challenge of balancing economic growth and environmental quality in industrial cities like Binzhou, particularly during rapid economic recovery.

Between 2019 and 2024, the interaction between GDP growth and compound pollution days in Binzhou evolved through distinct phases. In early 2020, despite a slight GDP increase compared to 2019 (amidst global pandemic-induced economic contractions), the number of compound pollution days decreased significantly. This was largely due to the reduction in anthropogenic pollution sources, such as decreased vehicle emissions and lower emissions from polluting enterprises, resulting from the early pandemic control measures [23]. The year 2021 saw a strong GDP rebound driven by economic stimulus policies. However, rapid recovery in energy-intensive sectors like chemicals and electrolytic aluminum sharply increased industrial emissions. Consequently, compound pollution days rose to 22-an increase of 9 days from 2020. After 2021, Binzhou strengthened environmental protection efforts, including restricting high-pollution industries in line with dual-carbon goals, which helped curb the growth of pollution days. The minor increase in compound pollution days during 2022–2023 coincided with economic recovery, higher industrial capacity utilization, and increased coal consumption, which raised pollutant emissions [22]. By 2024, Binzhou achieved significant economic growth while simultaneously improving air quality. This positive development was closely associated with the city’s investments in clean industries, technological upgrades in polluting enterprises, and the implementation of policies such as the “Action Plan for Continuous Improvement of Air Quality”. These measures demonstrate that through proactive structural adjustments and technological innovation, industrial cities can achieve sustainable development characterized by both economic growth and environmental improvement.

#### 3.3.2. Gray Relational Analysis of the Key Influencing Factors of Ecological Environment Air Quality

**Figure 9:**
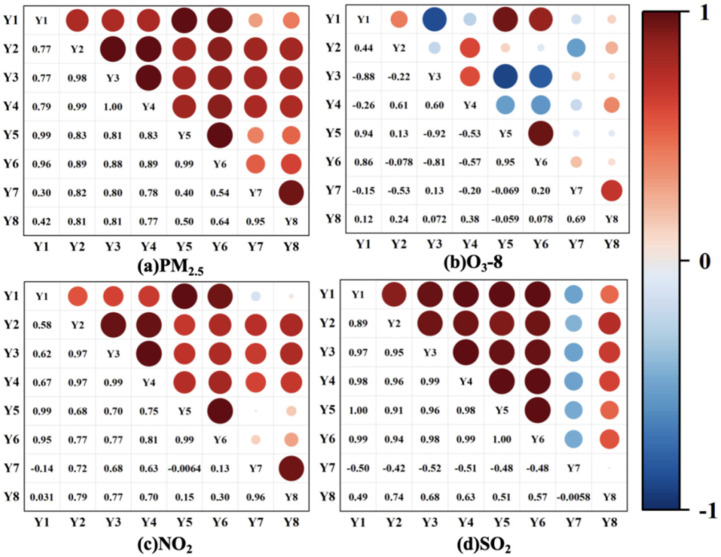
Gray correlation analysis of major pollutants in Binzhou city. Note: Y1 to Y8, respectively, represent regional GDP, industrial electricity consumption, population density, industrial energy consumption, agricultural output value, motor vehicle ownership, annual precipitation, and construction area of buildings.

The GRA results for PM_2.5_ show a clear structure of influencing factors, with correlation degrees ranging from 0.42 to 0.70 (Figure 9). Industrial electricity consumption (Y2) ranked highest (0.70), exceeding industrial energy consumption (Y4, 0.67) by 4.5%. This finding underscores the dominant role of industrial electricity use in driving PM_2.5_ emissions—likely due to Binzhou’s reliance on energy-intensive industries (e.g., electrolytic aluminum, chemicals), where electricity consumption is tightly linked to both primary PM_2.5_ emissions and precursor (SO_2_, NO_X_) release [22]. Population density (Y3, 0.67) and industrial energy consumption (Y4, 0.67) formed the second tier, with a negligible correlation difference (0.001). This reflects the synergistic effect of urbanization and industrialization: population agglomeration increases demand for industrial products and residential energy use, while industrial energy consumption directly emits PM_2.5_ [42]. Building construction area (Y8, 0.61) and motor vehicle ownership (Y6, 0.57) constitute the third tier, with a 6.7% correlation gap—indicating that infrastructure construction (a source of construction dust) has a stronger impact on PM_2.5_ than transportation emissions in Binzhou. This contrasts with studies in megacities like Beijing [6], where mobile sources are more prominent, highlighting the unique pollution structure of medium-sized industrial cities. Notably, agricultural output value (Y5, 0.54) and regional GDP (Y1, 0.53) exhibit weak correlations, with GDP ranking 7th. This unexpected result may reflect Binzhou’s industrial structure: the city’s GDP growth is driven by low-emission sectors (e.g., high-tech manufacturing) in recent years, while traditional agriculture (a potential source of dust and straw-burning emissions [43]) has a declining contribution to PM_2.5_. Annual precipitation (Y7, 0.42), the only natural factor, showed the lowest correlation, which is consistent with Wang et al. (2014) [29], who noted that anthropogenic emissions dominate PM_2.5_ in northern Chinese cities, outweighing the pollutant-scavenging effect of precipitation.

For O_3_-8 h, the correlation degrees of influencing factors were ranked as follows: industrial energy consumption (Y4, 0.92) exhibited the strongest correlation, followed by population density (Y3, 0.90), industrial electricity consumption (Y2, 0.88), motor vehicle ownership (Y6, 0.80), building construction area (Y8, 0.78), agricultural output value (Y5, 0.72), regional GDP (Y1, 0.71), and annual precipitation (Y7, 0.47). The exceptionally high correlation of industrial energy consumption (Y4) with O_3_-8 h (0.92)—far exceeding that in PM_2.5_ analysis—highlights the critical role of industrial precursor emissions (NO_X_, VOCs) in O_3_ formation [27]. Motor vehicle ownership (Y6, 0.80) ranked fourth, indicating that while transportation contributes to O_3_, its impact is muted compared to industrial sources-contrasting with studies in Baoding [12], where traffic-related NO_X_ dominates O_3_ precursor emissions. This difference underscores the need for region-specific O_3_ control strategies: Binzhou should prioritize industrial VOC/NO_X_ reduction, whereas larger cities may focus more on mobile sources. Annual precipitation (Y7, 0.47) showed the weakest correlation, confirming that meteorological factors such as temperature and solar radiation influence O_3_ concentrations more strongly than precipitation [21].

The GRA results for NO_2_ show a clear hierarchy of correlation degrees (0.46–0.77), with industrial electricity consumption (Y2, 0.77) ranking first. This aligns with Binzhou’s industrial structure: energy-intensive industries (e.g., coal-fired power plants, steel) use electricity for production processes and are major NO_X_ emitters, even with SCR/SNCR denitrification equipment [22]. Population density (Y3) and industrial energy consumption (Y4) tie for second (0.74), reflecting the synergistic effect of population agglomeration (increased transportation and residential energy use) and industrial activity on NO_2_ emissions [13]. Construction area showed a relatively strong correlation, suggesting that construction machinery emissions contribute more significantly to NO_2_ in Binzhou than on-road vehicles—a pattern not previously documented in other BTH corridor cities [3]. In contrast, agricultural output value (Y5, 0.59) and regional GDP (Y1, 0.58) demonstrated weak correlations. This likely reflects both the minimal NO_2_ emissions from agricultural activities and the ongoing decoupling between economic growth and high-emission industries [24]. Annual precipitation (Y7, 0.46) showed the weakest influence, supporting the established understanding that natural factors play a limited role in NO_2_ variations within human-dominated environmental systems [44].

For SO_2_, industrial electricity consumption (Y2, 0.72) showed the strongest correlation, confirming that industrial activity-particularly coal-fired electricity generation-serves as the primary SO_2_ source [22]. Population density (Y3, 0.72) and industrial energy consumption (Y4, 0.71) formed a second tier, with a correlation difference of less than 0.02. This reflects the synergy between urbanization and industrial coal use in driving SO_2_ emissions, as population concentration increases residential heating demand [29]. Building construction area (Y8, 0.68) and motor vehicle ownership (Y6, 0.61) rank in the middle, indicating moderate contributions from construction (e.g., coal use in on-site heating) and transportation (negligible for SO_2_, consistent with low sulfur in diesel fuel [13]). Agricultural output value (Y5, 0.57) and regional GDP (Y1, 0.56) show weak correlations, with GDP ranking seventh. This likely results from Binzhou’s ongoing transition toward low-sulfur industries such as high-tech manufacturing in its economic structure [24]. Annual precipitation (Y7, 0.52) is the weakest natural factor, but its correlation exceeds 0.5, suggesting that wet deposition has a minor but measurable influence on SO_2_ concentrations-a finding consistent with Zhang (2023) [28].

## 4. Conclusions

This study systematically analyzed the temporal variations, interactions, and key drivers of major air pollutants, particularly PM_2.5_ and O_3_, in Binzhou-an industrial and agricultural city situated within the Beijing–Tianjin–Hebei pollution transport corridor of the Yellow River Basin. Using high-resolution monitoring and socioeconomic data from 2019 to 2024, several key findings emerge. PM_2.5_ concentrations declined then rebounded, reaching their highest levels in winter owing to heating demand and stagnant atmospheric conditions. In contrast, O_3_ levels fluctuated but trended upward, peaking in summer as a result of photochemical activity. The number of compound pollution days fell by 50% over the study period, with most occurring in spring (March–May), identifying this season as a critical period for coordinated control. Seasonal correlation analysis revealed weak positive PM_2.5_-O_3_ associations in spring, summer, and autumn, strongest in summer, and a weak negative correlation in winter. These patterns reflect the influence of meteorological conditions and precursor emissions on pollutant interactions.

Gray relational analysis highlighted industrial electricity consumption and population density as dominant factors influencing PM_2.5_, while industrial energy consumption showed the strongest association with O_3_ formation. The study also observed a phased decoupling between economic growth and pollution levels. Meanwhile, the study contributes to the literature by delineating season-specific pollution dynamics and key socio-economic drivers in a medium-sized industrial city within a regional transport corridor, thereby addressing a critical research gap as the relationship between urban form, pollution, and their drivers can be distinctly different from those in megacities [45]. These results support the development of differentiated control strategies: prioritizing PM_2.5_ reduction in winter through clean heating and improved dispersion management, focusing on O_3_ precursors in summer, and implementing coordinated measures in spring. Future research should combine high-temporal-resolution monitoring with chemical transport modeling to better understand short-term pollution formation pathways and precursor sensitivity. Such approaches would strengthen the evidence base for synergistic pollution reduction and sustainable urban development in similar regional contexts.

## Figures and Tables

**Table 1:** The number of days with air quality grades and their Proportions in Binzhou city from 2019 to 2024.

Grade		2019	2020	2021	2022	2023	2024	Total
Good	Number	34	46	52	58	45	54	289
	Ratio	9.3%	12.6%	14.2%	15.9%	12.3%	14.8%	13.2%
Moderate	Number	173	190	193	195	171	190	1112
	Ratio	47.4%	52.1%	52.9%	53.4%	46.8%	52.1%	50.9%
Unhealthy for Sensitive Groups	Number	99	93	91	87	110	92	572
	Ratio	27.1%	25.5%	24.9%	23.8%	30.1%	25.2%	26.2%
Unhealthy	Number	44	24	21	21	28	24	162
	Ratio	12.1%	6.6%	5.8%	5.8%	7.7%	6.6%	7.4%
Very Unhealthy	Number	15	13	4	4	8	5	49
	Ratio	4.1%	3.6%	1.1%	1.1%	2.2%	1.4%	2.2%
Hazardous	Number	0	0	4	0	3	1	8
	Ratio	0.0%	0.0%	1.1%	0.0%	0.8%	0.3%	0.4%

**Table 2:** Average values of major pollutants in Binzhou city from 2019 to 2024 in different seasons.

Season		AQI	CO (mg/m^3^)	PM_2.5_ (μg/m^3^)	PM_10_ (μg/m^3^)	NO_2_ (μg/m^3^)	SO_2_ (μg/m^3^)	O_3_-8 (μg/m^3^)
Spring	Mean	96.86	0.79	40.84	88.51	31.42	15.74	128.07
	Median	89.00	0.70	38.00	73.00	30.00	11.00	132.00
Summer	Mean	102.42	0.69	27.20	49.22	19.46	9.72	159.60
	Median	93.00	0.60	28.50	56.00	24.50	13.00	144.00
Autumn	Mean	80.22	0.82	39.46	72.48	38.07	14.48	104.60
	Median	70.00	0.70	42.00	46.50	32.00	13.50	103.00
Winter	Mean	97.49	1.04	66.98	101.79	44.69	20.37	62.19
	Median	85.00	1.00	52.00	82.00	44.00	17.50	73.50

**Table 3:** The number of days of compound pollution in different seasons in Binzhou city from 2019 to 2024.

	Spring	Summer	Autumn	Winter	Total
2019	13	0	5	6	24
2020	3	0	6	3	12
2021	10	0	7	3	20
2022	2	0	4	1	7
2023	6	0	4	4	14
2024	5	0	4	3	12

## Data Availability

The pollutant data used in this study were obtained from the national public environmental data platform (https://www.aqistudy.cn/historydata/monthdata.php (accessed on 10 January 2025)). Socio-economic data were sourced from the Shandong Statistical Yearbook and the Binzhou Statistical Yearbook.
